# Designed Surface Residue Substitutions in [NiFe] Hydrogenase that Improve Electron Transfer Characteristics

**DOI:** 10.3390/ijms16012020

**Published:** 2015-01-16

**Authors:** Isaac T. Yonemoto, Hamilton O. Smith, Philip D. Weyman

**Affiliations:** J. Craig Venter Institute, Synthetic Biology and Bioenergy Group, 4120 Capricorn Lane, La Jolla, CA 92037, USA; E-Mails: isaac.yonemoto@gmail.com (I.T.Y.); hsmith@jcvi.org (H.O.S.)

**Keywords:** hydrogenase, ferredoxin, *Alteromonas macleodii*

## Abstract

Photobiological hydrogen production is an attractive, carbon-neutral means to convert solar energy to hydrogen. We build on previous research improving the *Alteromonas macleodii* “Deep Ecotype” [NiFe] hydrogenase, and report progress towards creating an artificial electron transfer pathway to supply the hydrogenase with electrons necessary for hydrogen production. Ferredoxin is the first soluble electron transfer mediator to receive high-energy electrons from photosystem I, and bears an electron with sufficient potential to efficiently reduce protons. Thus, we engineered a hydrogenase-ferredoxin fusion that also contained several other modifications. In addition to the *C*-terminal ferredoxin fusion, we truncated the *C*-terminus of the hydrogenase small subunit, identified as the available terminus closer to the electron transfer region. We also neutralized an anionic patch surrounding the interface Fe-S cluster to improve transfer kinetics with the negatively charged ferredoxin. Initial screening showed the enzyme tolerated both truncation and charge neutralization on the small subunit ferredoxin-binding face. While the enzyme activity was relatively unchanged using the substrate methyl viologen, we observed a marked improvement from both the ferredoxin fusion and surface modification using only dithionite as an electron donor. Combining ferredoxin fusion and surface charge modification showed progressively improved activity in an* in vitro* assay with purified enzyme.

## 1. Introduction

While a large amount of biofuel strain optimization effort has focused on the production of hydrocarbon [1,2] or alcoholic fuels [3,4], the efficiency of storing solar energy as organic molecules is hampered by energetically wasteful reactions including RuBisCo-mediated photorespiration [5] and CO_2_ release through fatty acid synthase. One alternative to increase efficiency of solar to chemical energy transfer is to reduce the number of biochemical steps involved; the most direct alternative is to use photosystem electrons to reduce protons to hydrogen gas [6].

H_2_ production in photosynthetic microorganisms is mediated by either [FeFe] or [NiFe] hydrogenases [7] or by nitrogenase [8]. However, inactivation of these enzymes by oxygen produced from photosystem II has required photobiological hydrogen production to avoid O_2_ using strategies such as inactivation of photosystem II [9] or spatiotemporal separation of the hydrogen producing reaction [8]. The net result of these strategies is that hydrogen is derived from solar energy stored as starches and energy is lost in the process. A photobiological hydrogen production system using an oxygen-tolerant hydrogenase from a non-photosynthetic microbe may allow hydrogen production to be directly coupled to the photosystem. 

Toward this goal of importing and improving hydrogenases in photosynthetic microbes, we chose to work with the *Alteromonas macleodii* “Deep Ecotype” [NiFe] hydrogenase, originally identified in the Global Ocean Survey [10] and subsequently found to exhibit modest oxygen tolerance [11,12]. Using work by others as a model [13,14], we designed two substitutions in the *A. macleodii* hydrogenase which presumably alter the electrochemical properties of the enzyme’s iron-sulfur clusters [12]. In the case of the *A. macleodii* hydrogenase, the combined double substitution, but neither of the single substitutions, resulted in a 3–4-fold improvement in proton reduction activity, as measured by a methyl viologen-mediated hydrogen evolution experiment. Hereafter, we refer to this doubly substituted enzyme as the generation-1 or “G1” enzyme.

We set out to further improve the G1 enzyme by directly coupling it to the cyanobacterial photosystem. Cyanobacterial photosystem delivers high-energy electrons to ferredoxin (PetF) carrying energy sufficient (midpoint potential < −400 mV) to reduce protons to hydrogen at pH 7.0 (−420 mV) [6]. Efforts by other researchers to engineer ferredoxin-coupled hydrogenase improved electron transfer by directly tethering ferredoxin to the hydrogenase [15], so our general strategy was to repeat this with our [NiFe] hydrogenase.

**Figure 1:**
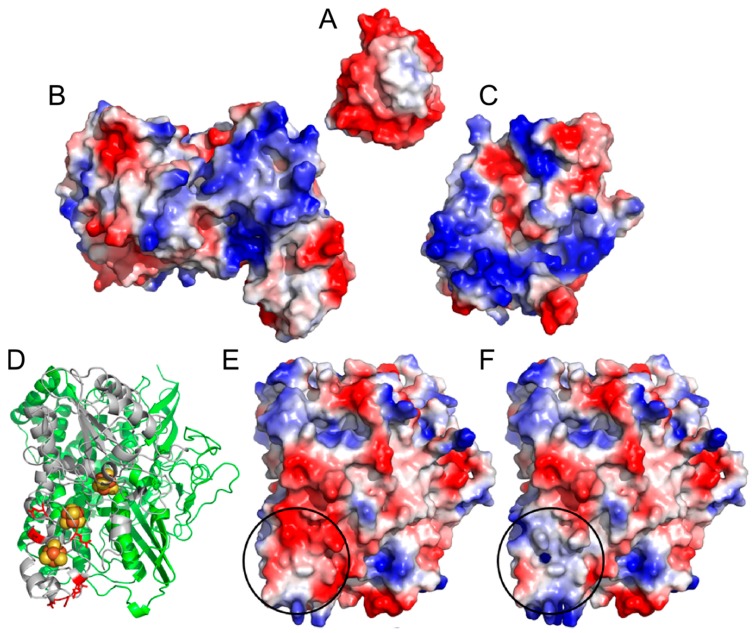
Electrostatic models of *S. elongatus* ferredoxin PetF (**A**); *Spinacia oleracea* PFOR (PDB: 1FNB) (**B**); Clostridial [FeFe] hydrogenase (PDB: 1FEH) (**C**); and a structural model of the *A. macleodii* hydrogenase small subunit (**D**). Negatively charged residues found near the docking site are colored red in the structural model. Electrostatic models of *A. macleodii* hydrogenase variants G1 (**E**) and G2 (**F**) in the same orientation as (**D**). Circled areas highlight the region near the distal Fe-S cluster. All models were obtained from the PDB where codes are given, or generated by the threading modeler Phyre. Charges were modelled using the default vacuum electrostatic package in PyMOL. In all electrostatic models, red is negatively charged and blue is positively charged.

We hypothesized that the negative charge on the hydrogenase small subunit would interfere with electron transfer from ferredoxin. While previous work showed that a covalent tether was sufficient to enhance [FeFe] hydrogenase activity [16], cyanobacterial ferredoxin is a negatively-charged molecule (Figure 1A) and [FeFe] hydrogenase features a highly positive cleft near the Fe-S cluster which accepts electrons, making it an attractive artificial electron acceptor (Figure 1B). Moreover, ferredoxin-NADPH reductase (FNR), a major natural electron acceptor of electrons from ferredoxin (Figure 1C), also features a positive patch near the active site. By contrast, the *A. macleodii* hydrogenase features negative residues near the distal Fe-S cluster (Figure 1D) and a corresponding negative patch (Figure 1E) near the desired interaction site. Polycationic molecules can interfere with electron transfer from the positively charged methyl viologen electron donor to the related *Thiocapsa roseopersecina* hydrogenase, presumably near the homologous negative patch [17]. Thus, a second goal of our project was substitution of these residues to neutralize this surface region and improve interaction of the hydrogenase with negatively-charged electron donors such as ferredoxin and dithionite (Figure 1F). In this work, we show the progressive improvement of hydrogenase activity with negatively charged electron donors as a result of both charge neutralization at the hydrogenase small subunit surface and direct tethering of ferredoxin to the hydrogenase small subunit.

## 2. Results and Discussion

### 2.1. Progressive Modification of Surface Residues

We sought to improve hydrogenase interaction with ferredoxin by substitution of the negatively-charged residues for neutrally-charged residues at the *A. macleodii* hydrogenase small subunit surface near the distal FeS cluster. Any such changes may lead to ambivalent effects: improvement of electron donor binding may be offset by defects in enzyme maturation or folding. However, dramatic surface modifications to improve enzyme biophysical properties have been reported with successful outcomes [18]. We therefore began with an ambitious design, “ALL+”, converting all negatively-charged residues near the distal iron-sulfur cluster to positively-charged residues. Although this enzyme was functional, it was severely impaired relative to the G1 enzyme, and was therefore abandoned. That it retained a small amount of activity gave promise for a more conservative redesign of the ferredoxin-interacting surface (see Supplementary Table S1).

**Figure 2:**
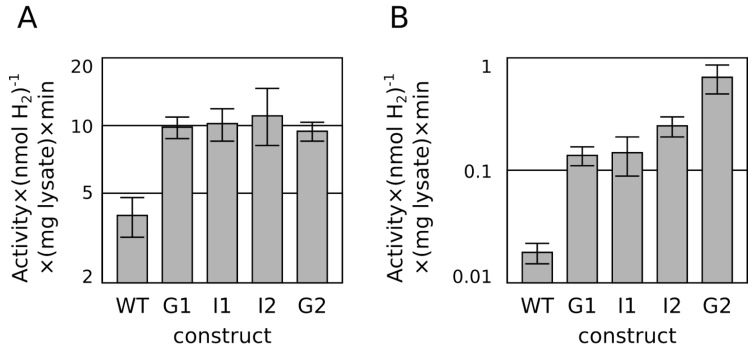
Methyl viologen-mediated (**A**) and methyl viologen-free (dithionite only) (**B**)* in vitro* hydrogen production assay from extracts of *E. coli* over-expressing the WT hydrogenase, G1 hydrogenase, and progressive substitutions to G2 hydrogenase (see Table 1 for sequence identities). Activities are plotted on a log scale over a 10-fold and 100-fold ranges, respectively to compare fold improvements.

A more modest series of surface residue modifications was pursued (see Table 1). Proceeding from the G1 enzyme, the double substitution E261Q, E268Q (“I1”) was constructed, followed by further substitution of E227Q (“I2”). Finally, the residue D231 was substituted as a histidine (D231H) following the substitution in a hydrogenase from *Azoarcus* sp. BH72 (YP_935309). While no appreciable difference could be measured between the variants with an* in vitro* hydrogen evolution assay using methyl viologen (Figure 2A), omission of the positively charged methyl viologen in favor of direct electron transfer from the anionic reducing agent dithionite (or possibly other soluble redox transfer species present in crude lysates) resulted in a dramatic 5-fold improvement in hydrogen evolution for the fully substituted 2nd generation “G2” enzyme compared to the G1 enzyme (Figure 2B).

**Table 1:** An alignment of the “surface patch” region of the *A. macleodii* hydrogenase and designed hydrogenases with select members of the hydrogenase small subunit family. The “ALL+” construct features five further substitutions outside of the alignment window. Substituted residues for each sequence are indicated red or blue with blue indicating substitution to positively charged residues.

Taxon	Sequence							
*Species*								
Accession	225	230	240	250	260	270	280	Name
Gammaproteobacteria *Alteromonas macleodii* AEA96483	FGESI	HDRCYR RPFF	EQRKFA KSFD	DEGAKNG WCL	FE-LGC KGPET	FNACAT VKWN	QGTSF PIE	“WT”
Engineered proteins *Alteromonas macleodii* AEA96483 derivatives	FGESI	CDRCYR RPFF	EQRKFA KSFD	DEGAKNG WCL	FE-LGC KGPET	FNACAT VKWN	QGTSF CIE	
	FGESI	CDRCYR RPFF	EQRKFA KSFD	DEGAKNG WCL	FQ-LGC KGPQT	FNACAT VKWN	QGTSF CIE	“I1”
	FGQSI	CDRCYR RPFF	EQRKFA KSFD	DEGAKNG WCL	FQ-LGC KGPQT	FNACAT VKWN	QGTSF CIE	“I2”
	FGQSI	CHRCYR RPFF	EQRKFA KSFD	DEGAKNG WCL	FQ-LGC KGPQT	FNACAT VKWN	QGTSF CIE	“G2”
	FGKSI	CRRCYR RPFF	KQRKFA KSFD	DEGAKNG WCL	FK-LGC KGPKT	FNACAT VKWN	QGTSF CIE	ALL+
	FGQSI	CHRCYR RPFF	EQRKFA KSF	GAK NGWCL	FQ-LGC KGPQT	FNACAT VKWN	QGTSF CIE	ΔDDE249
Cyanobacteria *Crocosphaera watsonii* EHJ10291	FRSFT	QTGCTR NMHF	SYKATT QDF	GQR TG-CL	FYDMGCRGP MT	HSSCNRI LWN	RVSS- KTR	
Actinobacteria *Collinsella tanakaei* ZP_08853311	FNQTV	HDNCPR RGHF	ENGEFV YQFG	SAEEAKG YCL	YP-LGC RGPQT	FTVCPVT RWN	QSVSW CVE	
Gammaproteobacteria *Beggiatoa alba* ZP_10114366	FGQTI	HDRCYR RPFY	DKGLFA DTFD	DEGAKQG WCL	YK-LGC KGPTT	YNACAT LKWN	DGVSF PIE	
Deltaproteobacteria *Desulfovibrio africanus str. “Walvis Bay”* YP_005053084	YGKTV	HEQCPR LKFF	EEDKFA PSFD	SEEARQG YCL	YQ-LGC KGPYT	YNNCPT AKFN	Q-VNW PVQ	
Betaproteobacteria *Azoarcus* sp.* BH72* YP_935309	ADQLV		HHGCSR NEFY	EFKASAE KPS	DLGCM	AHADCN LRPW	NGSGS CTS	
Alphaproteobacteria *Novosphingobium nitrogenifigens* ZP_08207308	ADHLV	HHACPK NEFY	EYKASA RALS	EMG CM	MEHLGC IGT-Q	AVGDCNI RPW	NGQGS CTR	

A third set of surface residue modifications was also constructed that was identical to the G2 except with the further deletion of three anionic residues that begin at amino acid number 249 (two aspartic acid residues followed by a glutamic acid residue, “DDE249”) (Table 1, Supplementary Table S1). While this showed similar initial activity to the G2 enzyme, this design was also abandoned due to difficulty reproducing the effect for longer hydrogen evolution times, which we speculate is a result of compromised protein stability. One final surface residue modification, E240Q, was also pursued, but this reduced activity (see Supplementary Table S1).

### 2.2. C-Terminal Truncation

Before fusing the hydrogenase with the cyanobacterial ferredoxin, the effect of truncation at the hydrogenase small subunit *C*-terminus needed to be tested. The *C*-terminus of [NiFe] hydrogenase has been suspected to be a hydrophobic membrane anchor [19], ultimately confirmed as a beta-staff interleaving in a beta sheet of the associated cytochrome c protein in *E. coli* [20]. In the case of the *A. macleodii* hydrogenase, this tail is shorter, highly charged, and unlikely to be a membrane anchor.

**Figure 3:**
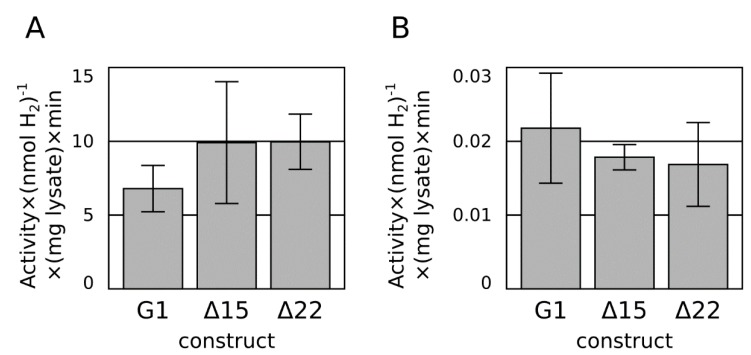
Methyl viologen-mediated (**A**) and methyl viologen-free (dithionite only) (**B**)* in vitro* hydrogen production assay from extracts of *E. coli* over-expressing the G1 hydrogenase and Δ15 and Δ22 truncations of the *C*-terminal tail.

Two designs for *C*-terminal truncation were tested. Based on homology to the newly released structure of *E. coli* hydrogenase-1 [20], a Δ15 construct was built to trim the *A. macleodii* hydrogenase small subunit *C*-terminus to the equivalent point where the membrane-embedded alpha-helix of the *E. coli* hydrogenase-1 begins. A more aggressive Δ22 construct was built to trim the *C*-terminal tail to the point where sequence conservation begins, and the polypeptide chain is exposed to solvent in multiple structures [20]. Neither truncation had an appreciable effect on the hydrogenase activity (Figure 3), so the Δ22 truncation strategy was employed moving forward.

### 2.3. Ferredoxin Fusion to Hydrogenase

Following a previously described design strategy [15], ferredoxin fusions were constructed by incorporating a 15-amino acid flexible linker at the *C*-terminus of the Δ22 G1 and G2 enzymes, followed by the ferredoxin, PetF from *S. elongatus* PCC 7942. Thus, the resulting fusion contained the *C*-terminus of the hydrogenase fused to the *N*-terminus of PetF. All four constructs (G1, G2, G1-PetF, and G2-PetF) were further modified with a Strep(II)-tag at the small subunit *C*-terminus and a His_6_ tag on the *N*-terminus of the HynL large subunit. After isolation from cell lysates using tandem immobilized metal affinity chromatography (IMAC) and Strep(II)-tag purification, protein concentrations for the four variants were adjusted to equal levels, and the enzymes were submitted to methyl viologen and methyl viologen-free hydrogen evolution assays.

Hydrogen evolution activity using methyl viologen was used as an estimate of total active enzyme and methyl viologen-free activities were normalized to this activity. The purified G2 enzyme exhibited improved relative methyl viologen-free activity compared to the purified G1 enzyme, consistent with previous results, but with a somewhat reduced effect, possibly due to elimination of nonspecific electron transfer agents in the lysate advantageous to G2 enzyme or the elimination of competing electron sinks that create an apparent lower activity of the G1 enzyme.

**Figure 4:**
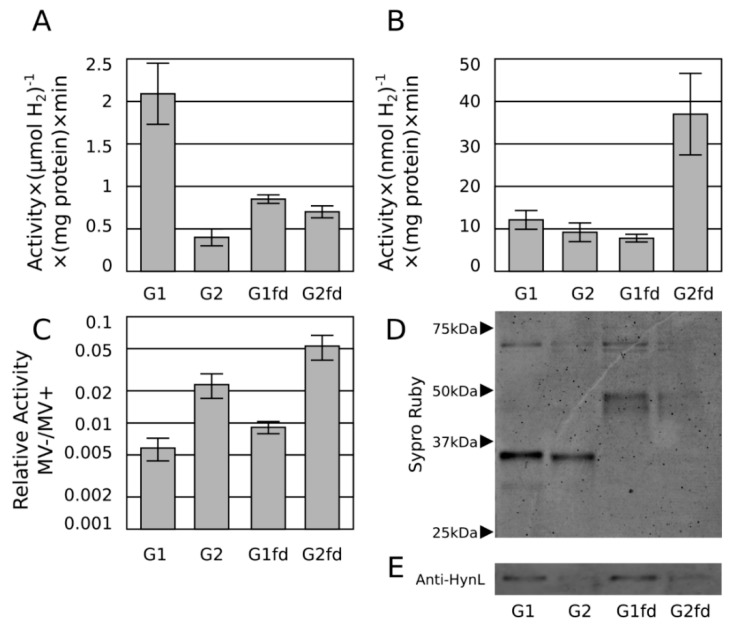
Methyl viologen-mediated* in vitro* hydrogen production assay (**A**) from tandem immobilized metal affinity chromatography (IMAC)/streptactin-purified G1 and G2 hydrogenases, and their respective ferredoxin fusions. *In vitro* H_2_ production of the same hydrogenases in the methyl viologen-free assay (dithionite only) (**B**) and expressed as the ratio of methyl viologen-free activity to activity in assays containing methyl viologen (**C**), plotted on a log scale over an 100-fold range to compare fold-changes*.* Sypro-ruby stained gel (**D**) and Anti-HynL western blot (**E**) of the same constructs from the same protein preparations presented in (**A**,**B**).

Also as predicted, the ferredoxin fusions exhibited approximately 2-fold improved methyl viologen-free activity compared to the unfused hydrogenase, suggesting enhanced electron transfer to the enzyme as a result of both modifications (Figure 4). This increased activity attributable to the fusion is not explained by differences in protein abundance as determined by Western blot (Figure 4).

### 2.4. Discussion

This study and our previous study [12] together show that the intrinsic activities of [NiFe] hydrogenase with respect to hydrogen production may be particularly amenable to improvement. Although they are traditionally considered to be inefficient at producing hydrogen, the heterodimer architecture of the uptake [NiFe] hydrogenases (among the [NiFe] hydrogenases) simplifies the enzyme complexity for iterative improvement. Unlike [FeFe] hydrogenases, substitutions can be generated and tested with ease in standard aerobic bench top environments; survival under those conditions is ultimately desired for enzyme function in oxygenic phototrophs. In the case of this study, we show that substitutions and protein-fusions to improve electron transfer characteristics can be rationally chosen, rapidly tested in model systems, and tolerated by the hydrogenase.

Unfortunately, the constructs produced in our study do not exhibit measurable hydrogen production* in vivo* when transferred to cyanobacteria. We believe this to be because of inefficient enzyme processing; previous studies show over ten-fold reduced specific activity loss between *E. coli* and *S. elongatus* [12]. Moreover, the complex assembly pathway for this class of enzymes may also require as-yet unexplored optimizations that will require more basic study. For example, in this study and in a previous work [21], we find weak suggestion that inefficient FeS cluster insertion may be responsible for low enzyme activities. Future work on this hydrogenase and variants should focus on improving active enzyme yields to enable the observation of* in vivo* hydrogen production in heterologous species, especially cyanobacteria.

We would also like to address the widespread use of methyl viologen as an electron transfer intermediate in hydrogen evolution studies for [FeFe] hydrogenases [16,22,23] as well as [NiFe] hydrogenases [13,14,24,25,26]. The positive charge of methyl viologen may not be an appropriate analog for all biological electron transfer agents, especially negatively charged ferredoxin, a likely candidate for biohydrogen purposes. Our results suggest that methyl viologen mediated reduction bypasses the canonical electron transfer entry point. There may also be effects as the dimeric nature of reduced methyl viologen [27] may make it an apt two-electron donor, with consequences in the special case of hydrogen reduction. Overall, care should be taken when choosing an electron mediator for* in vitro* hydrogenase activity assays, especially if protein surface modifications will be made. 

## 3. Experimental Section

### 3.1. Molecular Biology

A full list of primer names and sequences used for each plasmid in this study is available in the supplementary materials (Supplementary Table S2). Assembly notes for each plasmid including primers used can also be found in supplementary materials (Supplementary Table S3). Unless otherwise stated, plasmids were constructed using plasmid pCM012 [12] as a starting point. Plasmid pCM012 has four PTrc promoters placed throughout the hydrogenase gene cluster (assembled as pRC41-4 described below) and additionally modified with the H230C/P285C double substitution. To make the additional substitutions described here, desired mutations were constructed using primers encoding appropriate base pair changes to effect the corresponding substitutions in the *hynS* gene. Two amplicons: a 5' amplicon featuring the mutations introduced using a 3' primer, and a 3' amplicon featuring the mutations introduced using a 5' primer were combined with a BamHI/AgeI restriction digest of pCM012. These 3 DNA pieces featured ~20 bp overlap regions; and were assembled by Gibson isothermal assembly[28], transformed into DH10B, DH5alpha (NEB), or Epi300 (Epicentre) cells. Colonies were screened using PCR, and plasmids from positive clones were purified by alkaline lysis for sequence confirmation by Sanger DNA sequencing. Strep-tag and His-tag constructs were generated by using a two-piece Gibson isothermal assembly of the BamHI/AgeI-digested pCM012 [12], with a single amplicon featuring a long primer encoding the Strep-tag or the His-tag, as desired. Preparation of the enzyme featuring 10 substitutions was more complicated and is detailed in the supplementary materials (pIY042, pIY043, Supplementary Table S3). Preparation of constructs expressing fusions to PetF were generated using a similar strategy, except the 5' and 3' amplicons shared an artificial flexible linker in common, and the 3' amplicons were amplified from *S. elongatus* genomic DNA as template.

Plasmid pRC41 [29] was modified by adding three more P_Trc_ promoters throughout the cluster to supplement the existing P_Trc_ promoter at the 5' region of the gene cluster. The promoters were inserted as terminator-promoter units immediately preceding the following open reading frames (ORFs): *hynS*, *hypC*, and *hypD*. Additionally, the first two ORFs in the original pRC41, *orf1* and *cyt*, were removed from the expression construct because they were shown not to be important for heterologous expression in *E. coli* [29]. The terminator-promoter (TP) unit was constructed by amplifying the *rrnB* terminator from pTRC-NSI using primers T1 and T2, and the P_Trc_ promoter was assembled with oligonucleotides P1 and P2. The P_Trc_ promoter was additionally modified to include a strong ribosome binding site (5'-AAGAGGAGAAA). The resulting fragments were assembled using Gibson method into vector pUC19 that was previously digested with EcoRI and HindIII, and the resulting cloned plasmid was sequenced by Sanger DNA sequencing.

To insert the TP unit into various points within the hydrogenase cluster, a hierarchical assembly process was employed. First, the hydrogenase cluster was divided into two halves after the *hynL* open reading frame, and each half was assembled individually with the added TP units interspersed throughout the cluster. The first half was assembled as four overlapping PCR fragments with a TP unit inserted before each of the ORFs *orf2* and *hynS*. The first TP was amplified with primers AMR1-2 and AMR2; the region *orf2* to *hupH* was amplified by AMR3 and AMR12; the second TP unit was amplified with primers AMR13 and AMR14; and the final region of the first half which included *hynS* and *hynL* was amplified by primers AMR15 and AMR20-3. The fragments were purified (Qiagen PCR Cleanup kit) and assembled by Gibson method into pBR322 that was previously digested with BamHI and EcoRV. The second half of the hydrogenase gene cluster was amplified as four overlapping fragments and a TP unit was inserted before each of ORFs *hypC* and *hypD*. The TP before *hypC* was amplified with primers AMR1 and AMR22; the fragment containing *hypC-hypB* was amplified with primers AMR23 and AMR32; the second TP unit before *hypD* was amplified with primers AMR33 and AMR34; and the final fragment spanning *hypD to hypE* was amplified with primers AMR35 and AMR44. The four fragments were purified (QIAquick PCR Cleanup kit, Qiagen, Valencia, CA, USA) and were assembled into pTRC-NSI [30] that was digested with NdeI and BamHI. Each of the two halves was sequenced by Sanger DNA sequencing. The two halves were then amplified by PCR using primers AMR3-2RBSF and AMR20-4 for the first half and AMR21-2 and AMR44 for the second half, purified (QIAquick PCR Cleanup kit, Qiagen), and assembled using the Gibson method into vector pTRC-NSI that had been digested with EcoRI and NdeI. Resulting colonies were sequenced by Sanger sequencing to confirm correct assembly.

### 3.2. Hydrogenase Cleared Lysate Preparation

Hydrogenase crude lysates were prepared by transformation of BL21ΔH_4_ cells [31] with the respective hydrogenase expression plasmid. An overnight starter culture in 1 mL of LB + spectinomycin (40 μg·mL^−1^) was prepared; 100 μL of inoculum was then transferred to 25 mL Studier autoinducer media [32] with 100 μM NiCl_2_ and spectinomycin and incubated overnight under ambient air (16–20 h) at 30 °C, rotating 200 rpm. Overnight expression cultures were harvested by centrifugation at 3000× *g* for 25 min, resuspended in an additional 800 μL lysis buffer (10 mM tris, 1 mM DTT, 0.5 mM EDTA), sonicated on wet ice (Sonifier model 250, Branson, Danbury, CT, USA) for one minute on setting “4” with a 40% duty cycle. Sonicated cell matter was then centrifuged for 10 min, 16,800× *g*, at 4 °C, and the supernatant was removed to yield cleared lysate. Total protein content was assessed using 1× Bradford kit (Bio-Rad, Hercules, CA, USA) with BSA (NEB) as a control.

### 3.3. Tandem IMAC/Streptactin Hydrogenase Preparation

Purified hydrogenases were expressed identically as the crude lysates, except: 50 mL of autoinducer media supplemented with 100 μM NiCl_2_, 0.5% (*w*/*v*) alpha-lactose, and 0.01% (*w*/*v*) glucose was used for cell growth; NP buffer (50 mM Na_3_PO_4_, 100 mM NaCl, 1 mM 2-mercaptoethanol, pH 7.0) was used instead of lysis buffer; sonication was performed in two batches; and sonicated cell matter was centrifuged for 20 min instead of 10.

An IMAC spin column was prepared by applying 250 μL of TALON cobalt resin (Clontech, Mountainview, CA, USA) to an empty micro bio-spin column (Bio-Rad) and rinsing twice with deionized water and once with NP buffer. All spins except as noted were performed at 27× *g* for 10 s at 4 °C. The resulting cleared lysate was applied in two batches to the spin column; each batch was run through the column three times using 20 s spins. The columns were further washed using NP buffer (1 × 1 mL), NP buffer + 0.01% (*v*/*v*) Tween-20 (5 × 1 mL), and NP buffer + 10 mM imidazole (5 × 1 mL), and NP buffer (5 × 1 mL). Elution was achieved using two applications of LP buffer (NP buffer adjusted to pH 5.0). pH exchange was conducted by applying IMAC eluate to a 100 kDa Microcon spin column (YM-100, Millipore, Billerica, MA, USA) and exchanged in 2 × 500 μL of NP buffer.

Affinity purification for the Strep(II) tag was conducted by applying 200 μL of IMAC eluate to 100 μL of Strep-Tactin magnetic beads (Qiagen) in a 1.5 mL eppendorf tube (Denville, South Plainfield, NJ, USA), followed by 1 h incubation with end-over-end agitation at 4 °C. Beads were immobilized using magnetic separation and washed with NP buffer (4 × 1 mL), and eluted using 50 μL NP buffer + 10 mM biotin.

### 3.4. Hydrogenase Assay

Hydrogen evolution assays were performed as previously described [12]. Sample (cleared lysate or purified enzyme, 0.2 mL) was added to 1.7 mL of assay buffer containing 1.5 mL deionized water, 0.1 mL of 40 mg·mL^−1^ methyl viologen (Sigma, St. Louis, MO, USA) or deionized water, and 0.1 mL of 500 mM potassium phosphate, pH 7.0 (Life Technologies, Carlsbad, CA, USA). The samples were combined in 13 mL gastight vials and sealed using rubber septa (Sigma), and sparged under argon (Westair, San Diego, CA, USA) for 20 min to remove oxygen. After sparging, 0.1 mL of 2 M sodium dithionite (Sigma) was added by syringe anoxically. The resulting assay solution was a 1:10 dilution of sample containing 25 mM potassium phosphate, 8 or 0 mM methyl viologen, and 100 mM sodium dithionite, pH~7.0. For experiments with purified enzyme, samples were combined in 10 mL gastight vials, sparged under nitrogen, and otherwise treated similarly.

Cleared lysate samples were incubated for 2 h at 30 °C, followed by gas chromatog-raphy (CP-3800, Agilent Technologies, La Jolla, CA, USA) using a Fused Silica Molsieve 5A column (CP7537, Agilent Technologies) of 100 μL samples taken from the vial headspace.

Purified enzyme samples were first adjusted of protein concentration to 0.005 mg·mL^−1^, followed by dilution 1:10 in NP2 buffer. Methyl viologen-containing solutions were prepared by pipetting 200 μL of diluted sample in a total volume of 2 mL, sealing in an 11 mL vial with a rubber septum, sparging with nitrogen, then incubating for 20 h at 30 °C, followed by gas chromatography (6890N, Agilent Technologies) using a Fused Silica Molsieve 5A column (CP7537, Agilent Technologies) of 250 μL samples taken from the vial headspace. Methyl viologen-free solutions were prepared by pipetting 200 μL of diluted sample into a total volume of 1 mL, sealing in a 2 mL vial with an unused rubber septum, sparging, then incubating for 40 h at 30 °C, followed gas chromatography.

## 4. Conclusions

We have neutralized residues of the *A. macleodii* [NiFe] hydrogenase small subunit near the interface Fe-S cluster and have shown improved activity with a negatively charged electron donor as a surrogate for the negatively charged cyanobacterial ferredoxin. Furthermore, fusion of the cyanobacterial ferredoxin to the hydrogenase small subunit is tolerated and further improves electron transfer efficiency in combination with the charge neutralization. Overall, the *A. macleodii* hydrogenase has proven tolerant of a variety of substitutions and may be a good candidate for further improvement.

## Supplementary Materials

One supplementary file is included containing Table S1: Table of measured enzyme activities, Table S2: List of primers used in this study, Table S3: List of plasmids used in this study, and plasmid sequences listed in Genbank format. Supplementary materials can be found at http://www.mdpi.com/1422-0067/16/01/2020/s1.
